# Perceived and Personal Mental Health Stigma in Latino and African American College Students

**DOI:** 10.3389/fpubh.2018.00049

**Published:** 2018-02-26

**Authors:** Stacie Craft DeFreitas, Travis Crone, Martha DeLeon, Anna Ajayi

**Affiliations:** ^1^Social Sciences, University of Houston–Downtown, Houston, TX, United States

**Keywords:** mental health stigma, ethnic minority, Latino, African Americans, college students

## Abstract

Mental health stigma occurs when people have negative thoughts and beliefs of those with mental health illnesses or mental health treatment. Mental health stigma is related to an assortment of negative outcomes including discrimination in housing and employment, reduced usage of mental health services, and poor mental health outcomes. These implications may be particularly salient for ethnic minorities such as African Americans and Latinos who already suffer from other types of discrimination. This study examines perceived and personal mental health stigma in African American and Latino college students from a nontraditional university to help elucidate factors related to the development of mental health stigma. Students completed surveys concerning their stigma beliefs. African American students were found to have higher rates of mental health stigma than Latino students. Furthermore, anxiety about those with mental illness was related to greater mental health stigma for both groups. For African Americans, it was found that their perception of their ability to visibly identify those with mental illness was related to greater mental health stigma. These findings suggest that interventions to reduce mental health stigma in college students should target specific ethnic minority groups and focus on issues that are particularly salient to those communities.

## Introduction

Mental health stigma is a negative evaluation of those with mental illness or of mental health treatment. Mental health stigma can be conceptualized in a variety of ways, but in this paper two specific aspects of mental health stigma, perceived stigma and personal stigma, will be examined. Perceived stigma concerns negative attitudes where one believes that society as a whole holds about individuals with mental illness, while personal stigma focuses on one’s own beliefs about individuals with mental illness (1). Mental health stigma, particularly personal stigma, is important because those who hold stigma beliefs are less willing to obtain the needed treatment (1–9). Often due to stigma, individuals will avoid treatment until the disorder is nearly incapacitating. This avoidance is particularly pronounced in members of ethnic minority groups because they are less likely to seek mental health treatment than those of European Americans [e.g., Ref. (4, 10–12)].

Understanding the impact of mental health stigma on ethnic minority college students is particularly important in this growing population as almost half of college-aged students exhibit mental health problems (13). Despite these problems with mental health, less than a quarter of these students seek psychological treatment (13). The combination of high rates of mental health issues and low treatment begs the question “Why are college students not seeking treatment to improve their mental health?” Low rates of treatment seeking may be a result of high levels of mental health stigma.

Common stigma beliefs include that those with mental illness are dangerous (14), will not recover, and that their mental illness is their own fault (15). These types of beliefs can result in an assortment of negative consequences for those with mental illness such as low employment rates, poor and unsafe housing, as well as a reduction in the utilization of mental health care (7, 15–17). Further, we know that those who hold negative stigma beliefs may also have negative outcomes including the avoidance of treatment and poorer mental health (18–21). Mental health stigma among African American (22) and Latino college students (23) is related to negative attitudes about treatment. This study, therefore, seeks to examine what beliefs are related to mental health stigma and may play a role in the development of stigma for African American and Latino college students. By understanding the attitudes and beliefs that may influence stigma, we can begin to ameliorate the negative consequences for vulnerable populations. The goal of this study is to examine potential underlying beliefs related to stigma concerning those with mental illness and mental health treatment among Latino and African American college students in an effort to understand ways to reduce that stigma and in turn increase utilization.

### Mental Health Stigma and Ethnic Minorities

Mental health stigma is particularly important to understand among ethnic minorities because they may display higher levels of stigma toward those with mental illness [e.g., Ref. (14, 24)] and psychotherapy [e.g., Ref. (25, 26)] than among European Americans. A qualitative study suggested that African Americans were likely to feel embarrassment related to mental health problems and seeking treatment, stating that mental health stigma was a significant problem in their community (27). Another qualitative study found similar beliefs about embarrassment and that this embarrassment kept participants from seeking treatment (20). Among a community sample, it was noted that African Americans feared how others would react to them seeking mental health services (28). High levels of mental health stigma appear to be present in African American samples, indicating that stigma should be examined further in this population.

There is less research focusing on the relationship between Latinos and mental health stigma. A study conducted among Latino college students demonstrated that those with higher levels of stigma were less open to seeking mental health treatment (23). Similar findings are present in other populations of Latinos. Among Latinos caring for a relative with schizophrenia, mental health stigma was related to more depressive symptoms suggesting that stigma kept them from speaking about their relative to others and obtaining needed social support (19). An examination of older Latinos found that they reported a greater embarrassment concerning having mental illness than that of African Americans or European Americans, and that this shame may be related to beliefs about disappointing family (29). There is some evidence that mental health stigma is significantly involved in creating shame and impacting treatment seeking among Latinos, which suggests that further examination is needed.

Some studies have noted that ethnic minorities may not have higher levels of stigma than European Americans. For example, a study that compared African Americans and European Americans found that the European Americans in the study had more stigma beliefs, but it is important to note that 90% of this sample had prior psychological treatment (30). This high rate of treatment exposure makes this sample very unique and hard to generalize to the greater population. Further, a study found that Latina women held fewer stigma beliefs than European American and African American women, but Latinas also believed that mental health issues should not be discussed outside of the family (31). A study conducted among poor African American and Latina young women found that stigma is related to a lower desire to seek treatment, but only among immigrant women, not those who were US born (32). Corrigan et al. (33) suggested that ethnic minorities are less likely to hold stigma beliefs than European Americans. Unfortunately, this study, like many others, groups ethnic minorities together as if they are homogeneous. This grouping is a problematic trend in research, examining ethnic minorities, particularly when we know that there is much heterogeneity within and between ethnic groups. Because the relationship between ethnicity and mental health stigma is so complex, it warrants further examination.

Identifying the factors that are related to mental health stigma for ethnic minority college students is particularly important because of the implications of these beliefs on outcomes such as mental health usage, in addition to difficulties with employment and housing that may result from mental health issues that become exacerbated due to lack of treatment. If we can identify attitudes and beliefs that are related to the mental health stigma of African American and Latino college students, then they can be targeted in interventions designed to reduce mental health stigma and in turn result in better mental health outcomes overall.

### Why Do People Hold Mental Health Stigma Beliefs?

One of the most well-researched factors related to understanding mental health stigma for ethnic minorities is lack of knowledge. There is much research which suggests that ethnic minorities may have misinformation about mental illness. For example, African Americans are less likely than European Americans to believe in genetic causes for psychological disorders (34). Non-European American college students are also more likely to believe that a person’s psychological disorder is his or her own fault than European American college students (35). Research has also suggested that incorrect information about mental illness is even passed down through the generations in African American families (27). This misinformation about the cause of mental illness is a potential factor in perpetrating stigma beliefs (27, 34, 36). These ideas—i.e., not believing in biological causes of psychological disorder—are related to beliefs about how treatable psychological disorders are perceived to be and how likely those with mental illness will recover.

When examining what Latino populations understand about mental health, much research has focused on acculturation. Research suggests that greater acculturation is related to less stigma (37) and more willingness to seek mental health treatment (3, 4). This reduction in stigma may be due to acquiring knowledge of disorders as a part of becoming more familiar with American culture. Poor knowledge of mental health disorders is particularly problematic because the lack of knowledge about a mental illness often is related to individuals experiencing fear or anxiety about mental illness.

Interpersonal anxiety: much of the research that has examined the impact of interpersonal or intergroup anxiety on stigma and bias development has focused on ethnicity. Interpersonal anxiety is anxiety about interacting with someone perceived as different from oneself. Research suggests that many biases about other ethnic groups manifest and are maintained due to fears about interacting with that other group (38). For example, among European American medical students, racial bias was related to greater anxiety about interacting with African American patients and less willingness to treat them (39). There is evidence to suggest that anxiety functions in much the same way to produce mental health stigma. Those who had prior interactions with a person with schizophrenia were more willing to interact with those with schizophrenia in the future because they had more positive attitudes about those with schizophrenia (40). This suggests that contact with an individual with mental illness is related to less intergroup anxiety. An experiment that examined ways to reduce mental health stigma among British college students found that an intervention that reduced intergroup anxiety also resulted in less mental health stigma (41). This study did not examine a causal relationship between intergroup anxiety and mental health stigma, but offers additional support concerning the relationship between the two variables. It is of note that none of these studies were among ethnic minorities, but there is evidence to suggest that for African Americans, intergroup anxiety may be important in understanding their mental health stigma as they were more likely to want to limit contact with those having mental illness than Latinos or European Americans (42). It is important to examine whether interpersonal anxiety would be related to stigma beliefs for African Americans and Latinos as it is for other ethnic groups.

Interpersonal anxiety may also be related to the idea that those with mental illness are difficult to have a relationship with, so here the problem may not be fear, but that the person would demand too much attention and psychological energy. An examination of a Latino sample noted that individuals who desire social distance from those with mental illness were more likely to seek treatment (18). This suggests that there might be an unexpected relationship between avoiding relationships with those who have mental illness and stigma that warrants further examination.

Therapist efficacy: another factor that may be related to stigma beliefs for African American and Latino college students concerns beliefs about the capabilities or professional efficacy of the therapist. Some within the African American community fear the use of psychotropic medications (27). These fears are likely due to the mistrust of mental health professionals and those in the medical field in general. Among Latinos, beliefs about mental health treatment included that antidepressants were related to ideas about being “crazy” or associated with illegal drug use (43). In terms of psychotherapy, ethnic minorities reported being concerned about whether a therapist would be able to address their needs, perhaps due to discrimination and an unwillingness or inability to understand their life circumstances as a person of color (20). If therapists also hold stereotypes about ethnic minorities, how can they be trusted with treating their psychological problems? By contrast, among Latinos, mental health stigma was not related to an individual’s confidence in the ability of mental health professionals to treat disorders (23). These mixed findings suggest that examining beliefs about mental health professionals needs further examination.

### The Current Study

Overall, research suggests that exhibiting greater stigma toward those with mental illness can result in treatment underutilization for African Americans and Latinos [e.g., Ref. (20, 23)], and therefore it is important to better understand stigma to potentially improve mental health outcomes. We know that lack of knowledge may be related to anxiety and greater stigma, but it is not clear that merely increasing knowledge is responsible for stigma reduction in interventions (44). Further, there are connections between intergroup anxiety and concerns about the efficacy and effectiveness of mental health treatment that may play a role in mental health stigma development. This study examines the mental health perceptions of Latino and African American college students in an effort to understand what may fuel their stigma beliefs. Due to the mixed findings based upon ethnicity, we will not offer a hypothesis concerning whether Latinos or African Americans will manifest greater mental health stigma. However, because African Americans and Latinos may harbor different views concerning mental health, it is believed that different factors will be related to mental health stigma for each group. *Hypothesis 1*. For African Americans, intergroup anxiety will have a greater relationship with mental health stigma than for Latinos. *Hypothesis 2*. Due to mistrust between the African American community and mental health providers, issues such as the capabilities of mental health professionals (professional efficacy) and how treatable mental illnesses are will likely be more important for them than for Latinos. This study will also explore factors such as how the visibility of the disorder (which might relate to beliefs about the severity of mental illness) impacts mental health stigma, but offers no hypotheses on this issue.

## Materials and Methods

### Participants

Participants were students from an urban, commuter, 4-year college. Participants (*N* = 122) included African American (*N* = 47) and Latino (*N* = 75) college students with 78.7% identified as female. Of the African American sample, 93% were born in the United States, and 76% of the Latino sample was born in the United States. Of the Latinos born in the USA, 75% of them were first generation. Twelve percentage of the Latino sample were born in Mexico while another 12% were born in seven different countries across the Caribbean, South America, and Central America. The mean age was 26.36 (SD = 9.39) for the African American students and 21.79 (SD = 4.52) for the Latino students.

### Procedure

Students were recruited from psychology courses, primarily “Introduction to Psychology.” They were offered course credit or extra credit for completed participation. This study was carried out in accordance with the recommendations of Title 45 Code of Federal Regulations Part 46, Protection of Human Subjects, with written informed consent from all subjects. All subjects gave written informed consent in accordance with the Declaration of Helsinki. The protocol was approved by the Committee for the Protection of Human Subjects. Completion of the paper and pencil surveys took approximately 30 min and was conducted in groups of 7–20 students. Students’ identities were not connected to students’ data.

### Measures

#### Demographics

Demographics were obtained from participants in order to distinguish possible differences between groups based upon factors such as ethnicity and sex.

#### Mental Health Stigma

Mental health stigma was assessed with the Mental Health Stigma Scale (MHSS) (1). Typical questions on the MHSS include, “most people would willingly accept someone who has received mental health treatment as a close friend” and “I would willingly accept someone who has received mental health treatment as a close friend.” These questions measured *perceived stigma* and *personal stigma*, respectively. Participants noted their agreement with each statement on a 6-point scale. Higher scores indicate more stigmas. This scale was found to be reliable in previous research, perceived stigma, α = 0.89 and personal stigma, α = 0.78 (1). In the current study, reliability was evaluated separately for African Americans and Latinos. For African Americans, both perceived stigma (α = 0.85) and personal stigma (α = 0.70) were reliable. The scale was also reliable for Latinos (perceived stigma, α = 0.83, and personal stigma, α = 0.75).

Day’s Mental Illness Stigma Scale (45) assessed participants’ *specific stigma beliefs*. Gagged with a 6-point Likert scale, Day’s Mental Illness Stigma Scale measures attitudes toward persons with mental illness including interpersonal anxiety (feelings of anxiousness about interacting with a person with mental illness), relationship disruption (the degree to which the illness is perceived to disrupt relationships with others), hygiene (whether the illness is viewed as esthetically unpleasing), visibility (if the illness can supposedly be noticed or unnoticed by others), treatability (whether the illness is seen as treatable), professional efficacy (attitudes toward the ability of the therapist), and recovery (whether a person is expected to recover from mental illness). For example, a question measuring the perceived relationship disruption of someone with a mental illness asks, “I would find it difficult to trust someone with a mental illness.” All scales for this measure were reliable except for treatability. With an alpha of 0.66 for African Americans and 0.73 for Latinos, it showed an overall lower reliability, but because it was reliable for Latinos, it was included in analyses with caution. For African Americans, the reliability of the remaining scales ranged from 0.70 to 0.93, and for Latinos, the reliability ranged from 0.70 to 0.94.

The Marlowe–Crowne social desirability measure-Form C (MCSD-C) (46) was included to assess social desirability. This measure was used to help determine whether participants were answering truthfully or providing the socially desirable answer. The Form C is a shortened version of the MCSD measure. It includes 13 true/false questions. Sample items include “On few occasions, I have given up doing something because I thought too little of my ability” and “I have never deliberately said something that hurt someone’s feelings.” Some items are reverse scored. A higher score indicates greater indications of socially desirable responding. The measure was reliable for both African American (α = 0.85) and Latino participants (α = 0.71).

### Data Analysis Plan

Missing data were present in less than 1% of the data; therefore, the mean substitution was used for missing data. Due to the large number of predictors included in the primary regression analyses, a power analysis was conducted anticipating a small effect size of 0.2, with a desired statistical power being 0.8 and a probability of 0.05. This analysis demonstrated that the minimum sample size for the study would be 107 participants, which is below the 122 participants of this study.

In order to test the main study hypotheses, hierarchical regression was used. Histograms for each variable were examined to note the presence of skew, kurtosis, or non-normality. All variables were found to be acceptable. Residuals’ scatterplots were examined to ensure that there were no problems with homoscedasticity. There were no violations. Cook’s D and leverage were examined; it was noted that no variables were found to be influential. Tolerance was examined to identify cases of multicollinearity. No values approached tolerance scores of 0.2; therefore, we were not concerned about multicollinearity. As a result of this examination, analyses proceeded with data intact.

To examine the main study hypotheses, two separate hierarchical regression analyses were conducted. One regression focused on perceived stigma while the other focused on personal stigma as the outcome variable. The regression analyses were originally calculated including social desirability to determine whether this had an impact on the results. Social desirability was not correlated with perceived stigma or personal stigma for the entire sample and was not significant in the regression analyses; therefore, the reported regression analyses were conducted without social desirability as a control variable. For both regression equations, ethnicity, which was dummy-coded, was entered into the first step of the regression. In the second step, all of the specific stigma beliefs (intergroup anxiety, professional efficacy, treatability, recovery, relationship disruption, hygiene, and visibility) were entered. In the final step, interactions between the specific stigma beliefs and dummy-coded ethnicity were entered.

## Results

### Preliminary Analyses

Means and correlations for the main study variables can be found in Tables 1 and 2 respectively. An examination of the variable means using ANOVA found that there were no significant differences between African Americans and Latinos on any study variable other than perceived stigma with African Americans exhibiting significantly higher perceived stigma than Latinos, *F* (1, 120) = 4.32, *p* < 0.05. This finding suggests that African Americans are more likely than Latinos to believe that others hold stigma beliefs about those who are mentally ill. The difference between personal stigma for the two ethnic groups approached significance, *F* (1, 120) = 3.59, *p* = 0.060. Despite these differences, levels of mental health stigma were relatively low with the average response indicating agreement that those with mental illness should be treated like everyone else. Furthermore, social desirability was only significantly correlated with perceived stigma for African Americans, *r* = −0.30, *p* < 0.05, suggesting that African Americans who endorsed more perceived stigma were less like to provide socially desirable answers.

**Table 1:** Means and standard deviations for study variables.

	African Americans *N* = 47		Latinos *N* = 75	
	M	SD	M	SD
Personal stigma	2.37^+^	0.90	2.04^+^	0.93
Perceived stigma	3.87*	0.87	3.55*	0.79
Treatability	2.17	0.71	2.23	0.93
Rel. Disrup.	4.02	0.97	4.13	1.07
Hygiene	4.55	1.17	4.34	1.03
Anxiety	4.17	1.08	4.21	1.21
Visibility	4.06	1.03	4.06	1.21
Recovery	2.24	1.20	2.23	0.98
Prof. Eff.	2.36	0.91	2.18	1.06
Social. Des.	7.81	2.94	7.12	2.99

*p < 0.05, ^+^p = 0.060.

**Table 2:** Correlations for study variables.

	1	2	3	4	5	6	7	8	9	10
1. Personal stigma	–	0.14	0.44**	−0.59**	−0.58**	−0.66**	−0.27	0.53**	0.23	−0.23
2. Perceived stigma	0.13	–	−0.09	−0.10	0.14	−0.13	0.43**	−0.63	−0.42	−0.30*
3. Treatability	0.49**	0.11	–	–	−0.52**	−0.50**	−0.43**	0.75**	0.37*	−0.18
4. Rel. Disrup.	−0.66**	−0.20	−0.59**	–	0.60**	0.70**	0.34**	−0.72**	−0.24	0.20
5. Hygiene	−0.51**	−0.14	−0.52**	0.62**	–	0.66**	0.49**	0.56**	−0.07	−0.02
6. Anxiety	−0.68**	−0.17	−0.45**	0.65**	0.64**	–	0.46**	−0.64**	−0.21	0.17
7. Visibility	−0.33**	−0.25*	−0.21	0.30**	0.20	0.26*	–	−0.41**	−0.11	−0.12
8. Recovery	0.56**	0.25*	0.54**	−0.58**	−0.54**	−0.43**	−0.19	–	0.28	−0.20
9. Prof. Eff.	0.37**	0.03	0.44**	−0.25*	−0.19	−0.26*	−0.07	0.30**	–	−0.12
10. Social. Des.	−0.10	−0.11	−0.12	0.13	−0.01	0.10	0.08	−0.16	−0.11	–

*African Americans are above the diagonal and Latinos are below the diagonal*.

*p < 0.05, **p < 0.01.

### Primary Analyses

Two regression analyses were conducted. The first regression analyzed the relationship between specific stigma beliefs about those with mental illness and personal stigma (see Table 3). Overall, the variables explained 54% of the variance in personal stigma. Ethnicity was entered at the first step, but was only a marginally significant predictor of personal stigma, *t* = −1.90, *B* = −0.16, *p* = 0.060. In the second step, the specific stigma beliefs were entered. This step was significant, *R*^2^ = 0.54, *F* (1, 7) = 19.97, *p* < 0.001. Among the significant variables were ethnicity, *t* = −2.32, *B* = −0.14, *p* < 0.05, intergroup anxiety, *t* = −4.20, *B* = −0.31, *p* < 0.001, and professional efficacy, *t* = 2.22, *B* = 0.14, *p* < 0.05. These findings suggest that African Americans endorsed significantly more personal stigma than Latinos, that having high anxiety about engaging in a relationship with someone who is mentally ill is related to more personal stigma, and that having a positive view about therapists’ ability to treat mental illness was related to less personal stigma. The final step of the regression was not significant, indicating no significant interactions with ethnicity. This does not support *hypothesis 1* or *hypothesis 2* that intergroup anxiety, professional efficacy, or treatability would have a greater relationship with personal stigma for African Americans than for Latinos.

**Table 3:** Regression analyses examining relationship between ethnicity, specific stigma, and personal stigma.

		B	SE B	95% CI B	β	t	ΔR^2^	ΔF
**Model**								
1							0.03	3.61
	Ethnicity	−0.16	0.09	[−0.33, 0.01]	−0.17	−1.90		

2							0.54***	19.97
	Ethnicity	−0.14*	0.06	[−0.26, −0.02]	−0.15*	−2.32		
	Treatability	−0.01	0.10	[−0.21, 0.18]	−0.01	−0.14		
	Rel. Disrup.	−0.18*	0.09	[−0.36, −0.10]	−0.20*	−2.06		
	Hygiene	−0.05	0.08	[−0.20, 0.11]	−0.05	−0.59		
	Anxiety	−0.31***	0.07	[−0.46, −0.16]	−0.39***	−4.20		
	Visibility	−0.05	0.05	[−0.15, 0.06]	−0.06	−0.84		
	Recovery	−11	0.08	[−0.04, 0.27]	0.13	1.48		
	Prof. Eff.	0.14*	0.06	[0.02, 0.27]	0.15*	2.22		

3							0.03	0.933
	Ethnicity	−0.15*	0.06	[−0.27, −0.03]	−0.15*	−2.40		
	Treatability	0.03	0.12	[−0.21, 0.27]	0.03	0.24		
	Rel. Disrup.	−0.15	0.10	[−0.34, 0.05]	−0.16	−1.50		
	Hygiene	−0.07	0.08	[−0.22, 0.09]	−0.08	−0.85		
	Anxiety	−0.35***	0.04	[−0.52, −0.19]	−0.44	−4.23		
	Visibility	−0.01	0.06	[−0.11, 0.14]	0.02	0.20		
	Recovery	0.10	0.09	[−0.07, 0.27]	0.11	1.13		
	Prof. Eff.	0.16*	0.07	[0.02, 0.29]	0.17*	2.24		
	EthXTreatability	−0.05	0.12	[−0.29, 0.19]	−0.04	−0.38		
	EthXRel.Disrup.	−0.05	0.10	[−0.25, 0.14]	−0.06	−0.55		
	EthXHygiene	0.12	0.08	[−0.04, 0.27]	0.13	1.47		
	EthXAnxiety	0.05	0.08	[−0.12, 0.21]	0.06	0.56		
	EthXVisibility	−0.10	0.06	[−0.23, 0.02]	−0.13	−1.62		
	EthXRecovery	0.12	0.09	[−0.06, 0.29]	0.13	1.34		
	EthXProf. Eff.	−0.03	0.07	[−0.16, 0.11]	−0.03	−0.38		

*p < 0.05, **p < 0.01, ***p < 0.001.

When examining perceived stigma, the collection of constructs explained 24% of the variance, *R*^2^ = 0.24, *F* (1, 7) = 3.11, *p* < 0.01 (see Table 4). In the first step, ethnicity was significantly related to perceived stigma, *t* = −2.08, *B* = −0.16, *p* < 0.05. Similar to the finding above, African Americans exhibited greater levels of perceived stigma than Latinos. The second step of the regression was not significant. The final step, which included the interaction terms, was significant and contributed an *R*^2^ change of 0.16. Among the significant predictors were intergroup anxiety, *t* = −2.15, *B* = −0.22, *p* < 0.05, visibility, *t* = 2.10, *B* = 0.16, *p* < 0.05, and the interaction between visibility and ethnicity, *t* = −3.85, *B* = −0.30, *p* < 0.001. Those who had less intergroup anxiety concerning people with mental illness were less likely to believe that others held stigma beliefs about the mentally ill. This does not support *hypothesis 1* or *hypothesis 2* that intergroup anxiety, professional efficacy, or treatability would have a greater relationship with stigma for African Americans than for Latinos because the ethnicity interactions with these variables were not significant. Because the interaction between visibility and ethnicity was significant, the direct effect of visibility was not interpreted.

**Table 4:** Regression analyses examining relationship between ethnicity, specific stigma, and perceived stigma.

		*B*	SE*B*	95% CI*B*	β	*t*	Δ*R*^2^	Δ*F*
**Model**								
1							0.04	4.34*
	Ethnicity	−0.16*	0.08	[−0.31, −0.01]	−0.19*	−2.08		

2							0.05	0.894
	Ethnicity	−0.13	0.08	[−0.29, 0.02]	−0.16	−1.74		
	Treatability	−0.06	0.13	[−0.31, 0.19]	−0.06	−0.48		
	Rel. Disrup.	−0.13	0.12	[−0.36, 0.10]	−0.16	−1.13		
	Hygiene	0.13	0.10	[−0.07, 0.33]	0.17	1.33		
	Anxiety	−0.12	0.10	[−0.31, 0.07]	−0.17	−1.26		
	Visibility	0.04	0.07	[−0.10, 0.18]	0.05	0.53		
	Recovery	0.06	0.10	[−0.14, 0.25]	0.07	0.55		
	Prof. Eff.	−0.02	0.08	[−0.19, 0.14]	−0.03	−0.27		

3							0.16	3.11**
	Ethnicity	−0.14	0.07	[−0.28, 0.01]	−0.16	−1.85		
	Treatability	−0.004	0.15	[−0.29, 0.29]	−0.004	−0.03		
	Rel. Disrup.	−0.09	0.12	[−0.33, 0.15]	−0.11	−0.76		
	Hygiene	0.09	0.10	[−0.10, 0.28]	0.12	0.96		
	Anxiety	−0.22*	0.10	[−0.42, −0.02]	−0.31*	−2.15		
	Visibility	0.16*	0.08	[0.01, 0.31]	0.22*	2.10		
	Recovery	0.02	0.11	[−0.19, 0.23]	0.03	0.18		
	Prof. Eff.	0.00	0.09	[−0.17, 0.17]	−0.001	−0.005		
	EthXTreatability	−0.06	0.15	[−0.35, 0.24]	−0.06	−0.38		
	EthXRel.Disrup.	0.07	0.12	[−0.17, 0.30]	0.08	0.55		
	EthXHygiene	−0.06	0.10	[−0.25, 0.13]	−0.08	−0.66		
	EthXAnxiety	0.18	0.10	[−0.02, 0.38]	0.25	1.77		
	EthXVisibility	−0.30***	0.08	[−0.45, −0.14]	−0.41***	−3.85		
	EthXRecovery	0.17	0.11	[−0.04, 0.38]	0.21	1.57		
	EthXProf. Eff.	−0.02	0.09	[−0.19, 0.24]	−0.03	−0.28		

*p < 0.05, **p < 0.01, ***p < 0.001.

**Figure 1:**
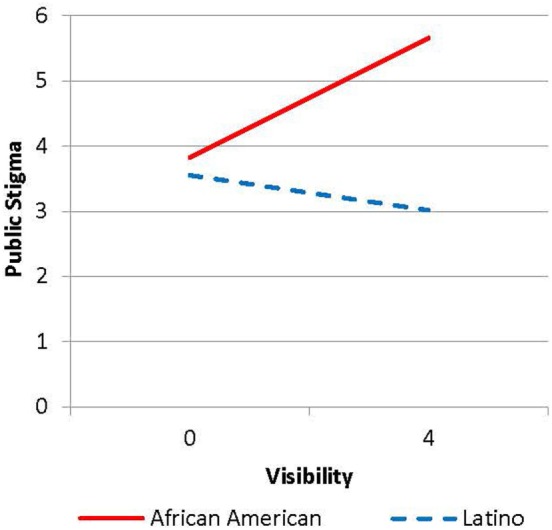
Simple slopes for relationship between visibility and ethnicity.

The interaction between visibility and ethnicity was examined using the simple slopes method [(47); see Figure 1]. Examination suggested that for African Americans, their perceptions of being able to identify those with mental illness more easily were related to greater beliefs in stigma, *t* = 3.46, *p* < 0.001. The relationship was not significant for Latinos, *t* = −1.80, *p* = 0.075.

## Discussion

Past research suggests that mental health stigma beliefs may be related to anxiety about interacting with those with mental health stigma [e.g., (40)] and to beliefs that mental health professionals cannot treat these disorders [e.g., (20)], but this has not been well examined in an African American and Latino college population. This study adds to this research by examining these factors in an African American and Latino college sample in order to better understand the evolution of mental health stigma beliefs. As suggested by previous research [e.g., (42)], African Americans had greater perceived and personal stigma than Latinos. Yet, both study hypotheses, that for African Americans, interpersonal anxiety, professional efficacy, and treatability would have stronger relationships with mental health stigma than for Latinos, were not supported. However, as expected, the mental health stigma of African American and Latino college students was related to different factors. For African Americans, believing that they were able to identify those with mental illness was related to greater beliefs in perceived stigma. For both ethnic groups, having high anxiety about interacting with someone who is mentally ill was related to more personal stigma and more perceived stigma. In addition, having a positive view about therapists’ ability to treat mental illness and believing that a mental illness would not be disruptive to a relationship were also related to less personal stigma.

For African Americans, the relationship between greater visibility of those with mental health problems and stigma may be due to their perceptions of the severity of those problems. It is likely that African Americans see psychological disorders as highly visible because they are considering only severe mental health issues. This belief is supported by previous findings which suggested that for many African Americans, having mental health problems means that your problems are significant enough to warrant inpatient hospitalization (27) and that if you are seeking treatment, you must have a serious mental illness (20). Equating mental illness and psychological treatment only with severe mental illness is likely to result in greater stigma toward the mentally ill. In addition, familiarity with schizophrenia is actually associated with increased stigma in the African American population, contrary to beliefs that exposure and familiarity with mental illnesses reduce stigmatizing beliefs and behaviors (48). Believing that psychological disorders are highly visible and acknowledging only severe mental illness can lead to increased social distance from those with mental illness. Due to the potential impact of beliefs about the severity of disorders on stigma, educating people about the range in the severity of mental health problems and the treatability of mental problems before they become debilitating is essential.

According to the current study, African American and Latino college students who believe that they can have a positive relationship with someone with a disorder and that therapists can effectively treat mental illness are less likely to have personal stigma. Therefore, believing that mental illness is malleable and treatable is related to having fewer negative beliefs about mental illness. When African American and Latino undergraduates believe that the state of having a mental disorder is temporary, then they are less likely to have stigma beliefs about it. These results are similar to those found primarily among a European American and Asian sample in which more positive attitudes concerning treatment were related to less perceived stigma (49). African Americans and Latinos also have less stigma when they believe that a psychological disorder will not be disruptive to their relationship with someone. They are likely to anticipate less disruption when assuming that the disorder is less severe. As noted above, if mental illness is viewed as less severe, then one is less likely to stigmatize those with mental illness and more open to relationships with them.

Because people of ethnic minority status are often at risk for not receiving adequate mental health care given their lower socioeconomic status and their distrust of the mental health system (50), intervening with this population is key to improving mental health outcomes. For example, African Americans and Latinos are less likely to seek treatment [e.g., Ref. (51, 52)], less likely to maintain treatment once they begin (53), and more likely to delay treatment until symptoms are debilitating (12). Much of this hesitation may be due to a negative history with the medical field, but it is also possible that stigma plays a role in these negative outcomes for ethnic minorities. Further, ethnic minorities may be experiencing the negative impact of mental health stigma at a more significant rate than European Americans.

### Clinical Applications

The most significant factor in predicting personal stigma was interpersonal anxiety as well as a strong predictor of perceived stigma and therefore an important target for interventions. Both African American and Latino college students were more likely to experience and perceive greater stigma if they were anxious about interacting with those with mental illness. Because anxiety has such a strong relationship with stigma, this is worth more consideration. College-counseling centers should offer programs that focus on reducing mental health stigma by targeting the reduction of interpersonal anxiety for African American and Latino college students. Contact programs expose individuals to those with mental illness or other groups considered to be different from them in an effort to reduce stigma (54, 55). As noted above, imagined positive interactions with a person with schizophrenia reduced anxiety for such future interactions among British college students and increased their belief that they would be willing to have contact with a person with schizophrenia (41). Because less familiarity with mental disorders is related to more fear concerning them (35), interventions that increase familiarity will likely reduce this fear. However, this is not a simple remedy as research suggests that African Americans may continue to hold stigma beliefs despite contact with individuals suffering from mental illness (48, 56). For ethnic minorities, it may be particularly important to include interactions with a person who has mental illness from their ethnic group. This similarity will assist in alleviating any potential distrust that may arise when dealing with the mental health system. Further, because this study suggests that ethnic minorities, particularly African Americans, may only expect mental illness to be severe, it is important to include interactions with those who have mild or moderate symptoms who can demonstrate the ability to live a full life with a mental illness.

Education about mental illnesses, mental health providers, and other issues relating to mental health is integral to the erosion of mental health stigma. However, education programs cannot be one-size-fits-all due to the differing cultural attitudes and stereotypes toward mental illnesses. A focus group of African Americans expressed their desires that mental health information and services should be best provided in a reassuring manner, from a credible source, and be easily accessible (24). The perception of credibility and accessibility can be increased, not only by having the information provided by doctors or mental health researchers but also by having the providers be respected public figures who look similar to them and who speak in layman’s terms, avoiding advanced medical terminology. Respected members of the university community may be ideal choices for helping to educate students about mental health issues. Spiritual or religious leaders may also be ideal for this role. In addition, the manner in which this information is delivered is important as well. These types of interventions that are specific to particular ethnic communities are essential to the reduction of stigma.

African American’s and Latino’s help-seeking intentions are diminished by their perception of peers’ mental health stigma (4, 25), and African Americans are especially impacted by their perceptions of their family’s attitudes (25). To better combat this, mental health education campaigns targeting African Americans and Latinos must better target their areas of need by including family and friends. Conducting workshops during parent weekends or among residence hall groups may help with building a community that is more knowledgeable about mental health. This more communal approach may be an integral part of this type of intervention for ethnic minorities.

Finally, training programs for mental health workers need to educate their students about mental health stigma for all individuals, but particularly for clients of color. If the next generation of mental health providers is able to address stigma concerns with their clients early, they may be able to build better rapport and facilitate longer treatment which would lead to improved mental health outcomes. Therapists must be able to understand the potential sources of stigma which would allow them to challenge stigma beliefs, therefore reducing stigma in clients.

### Limitations

Despite the deeper evaluation of the types of beliefs that are related to mental health stigma in African American and Latino populations, this study included limitations of note. One limitation of the sample in this study was the relatively low levels of stigma. This could be due to the fact that the study was primarily female—females are less likely to have stigma beliefs than males (14)—and they were college students taking at least one psychology course. Their knowledge of psychology may have led them to be less biased. Future studies should target a college group who has not been educated in psychological issues and a sample that has a more equal gender divide. In addition, this sample is a convenience sample of nontraditional college students which limits generalization. Furthermore, because of the research design, causation cannot be determined. A longitudinal or experimental study would be better able to elucidate the direction of effect and provide more confidence that targeting factors such as anxiety will lead to reduced stigma. Finally, in this study, the Latino sample is a collection of students of varying nationalities and generational statuses. This sample may be more heterogeneous than is suggested by these findings. Future research should examine specific Latino populations.

Future research should continue to explore causes of stigma in ethnic minority groups. Research on Asian American and Latino college students is particularly sparse [noted exception (3)]. Further, this study did not look at religious beliefs and spirituality as possible factors that may be related to stigma and/or impact treatment seeking. Previous research suggests that such beliefs warrant future study [e.g., (27)].

Developing mental health stigma reduction programs specifically targeting ethnic minority groups, particularly college students, should be a primary goal of mental health practitioners due to these groups’ reduced usage of mental health services. Exposing ethnic minorities to individuals with mental health problems of their own ethnicity, allowing one-on-one interaction, and targeting their specific stigma areas (i.e., interpersonal anxiety, severity of mental illness, and treatability of mental illness) may lead to reduced stigma and in turn better mental health outcomes overall.

## Ethics Statement

This study was carried out in accordance with the recommendations of the Protection of Human Subjects policy administered by the Committee for the Protection of Human Subjects with written informed consent from all subjects. All subjects gave written informed consent in accordance with the Declaration of Helsinki. The protocol was approved by the UHD Committee for the Protection of Human Subjects.

## Author Contributions

SD developed the study. She planned and organized all data collection, analyzed the data, and wrote about 50% of the paper. TC wrote about 40% of the paper and assisted with major revisions. MD and AA were students while working on this project. Together, they wrote the remaining 10% of the paper and assisted with data collection.

## Conflict of Interest Statement

The authors declare that the research was conducted in the absence of any commercial or financial relationships that could be construed as a potential conflict of interest. The reviewer LH and the handling editor declared their shared affiliation.

## References

[1] Eisenburg D Downs M Golberstein E Zivin K. Stigma and help seeking for mental health among college students. Med Care Res Rev (2009) 66:522–41. 10.1177/1077558709335173 19454625

[2] Bathje GJ Pryor JB The relationships of public and self-stigma to seeking mental health services. J Mental Health Couns (2011) 33(2):161–77. 10.17744/mehc.33.2.g6320392741604l1

[3] Cheng H Kwan K Sevig T. Racial and ethnic minority college students’ stigma associated with seeking psychological help: examining psychocultural correlates. J Couns Psychol (2013) 60(1):98–111. 10.1037/a0031169 23356468

[4] Cheng H McDermott RC Lopez FG Mental health, self-stigma, and help-seeking intentions among emerging adults: an attachment perspective. Couns Psychol (2015) 43(3):463–87. 10.1177/0011000014568203

[5] Clement S Schauman O Graham T Maggioni F Evans-Lacko S Bezborodovs N What is the impact of mental health-related stigma on help-seeking? A systematic review of quantitative and qualitative studies. Psychol Med (2015) 45(1):11–27. 10.1017/S0033291714000129 24569086

[6] Jennings KS Cheung JH Britt TW Goguen KN Jeffirs SM Peasley AL How are perceived stigma, self-stigma, and self-reliance related to treatment-seeking? A three-path model. Psychiatr Rehabil J (2015) 38(2):109–16. 10.1037/prj0000138 25844914

[7] Sharac J McCrone P Clement S Thornicrogt G. The economic impact of mental health stigma and discrimination: a systematic review. Epedimiol Psichiatr Soc (2010) 13(3):223–32. 10.1017/S1121189X00001159 21261218

[8] US Department of Health and Human Services. Mental Health: A Report of the Surgeon General. Rockville, MD: US Department of Health and Human Services (1999).

[9] Vogel DL Wade NG Haake S Measuring the self-stigma associated with seeking psychological help. J Couns Psychol (2006) 53:325–37. 10.1037/0022-0167.53.3.325

[10] De Luca SM Blosnich JR Hentschel EW King E Amen S Mental health care utilization: how race, ethnicity and veteran status are associated with seeking help. Community Ment Health J (2015) 52(2):174–9. 10.1007/s10597-015-9964-3 26659853

[11] Dobalian A Rivers PA. Racial and ethnic disparities in the use of mental health services. J Behav Health Serv Res (2008) 35(2):128–41. 10.1007/s11414-007-9097-8 18074230

[12] Satcher DS Executive summary: a report of the surgeon general on mental health. Public Health Rep (2000) 115:89–101. 10.1093/phr/115.5.489 10968589 PMC1308561

[13] Blanco C Okuda M Wright C Hasin DS Grant BF Liu S Mental health of college students and their non-college-attending peers: results from the national epidemiologic study on alcohol and related conditions. Arch Gen Psychiatry (2008) 65(12):1429–37. 10.1001/archpsyc.65.12.1429 19047530 PMC2734947

[14] Corrigan PW Watson AC. The stigma of psychiatric disorders and the gender, ethnicity and education of the perceiver. Community Ment Health J (2007) 43:439–57. 10.1007/s10597-00709084-9 17876705

[15] Watson AC Eack SM Oppression and stigma and their effects. In: Heller NR Gitterman A, editors. Mental Health and Social Problems: A Social Work Perspective. New York: Routledge (2011). p. 21–43.

[16] Corrigan PW Kleinlein P The impact of mental illness stigma. In: Corrigan PW, editor. On the Stigma of Mental Illness: Practical Strategies for Research and Social Change. Washington, D.C: American Psychological Association (2005). p. 11–44.

[17] Livingston JD Boyd JE. Correlates and consequences of internalized stigma for people living with mental illness: a systematic review and meta-analysis. Soc Sci Med (2010) 71(12):2150–61. 10.1016/j.socscimed.2010.09.030 21051128

[18] Interian A Ang A Gara MA Link BG Rodriguez MA Vega WA. Stigma and depression treatment utilization among Latinos: utility of four stigma measures. Psychiatr Serv (2010) 61(4):373–9. 10.1176/ps.2010.61.4.373 20360276 PMC3222155

[19] Magaña SM García JR Hernández MG Cortez R Psychological distress among Latino family caregivers of adults with schizophrenia: the roles of burden and stigma. Psychiatr Serv (2007) 58(3):378–84. 10.1176/appi.ps.58.3.378 17325112 PMC2396526

[20] Thompson VL Bazile A Akbar M African Americans’ perceptions of psychotherapy and psychotherapists. Prof Psychol Res Prac (2004) 35(1):19–26. 10.1037/0735-7028.35.1.19

[21] Vega WA Rodriguez MA Ang A. Addressing stigma of depression in Latino primary care patients. Gen Hosp Psychiatry (2010) 32:182–91. 10.1016/j.genhosppsych.2009.10.008 20302993

[22] Masuda A Anderson PL Edmonds J Help-seeking attitudes, mental health stigma, and self-concealment among African American college students. J Black Stud (2012) 43(7):773–86. 10.1177/0021934712445806

[23] Mendoza H Masuda A Swartout KM Mental health stigma and self-concealment as predictors of help-seeking attitudes among Latina/o college students in the United States. Int J Adv Counsel (2015) 37(3):207–22. 10.1007/s10447-015-9237-4

[24] Mishra SI Lucksted A Gioia D Barnet B Baquet CR. Needs and preferences for receiving mental health information in an African American focus group sample. Community Ment Health J (2009) 45(2):117–26. 10.1007/s10597-008-9157-4 18633704 PMC3618894

[25] Barksdale CL Molock SD Perceived norms and mental health help seeking among African American college students. J Behav Health Serv Res (2008) 36(3):285–99. 10.1007/s11414-008-9138-y 18668368

[26] Brown C Conner KO Copeland V Grote N Beach S Battista D Depression stigma, race, and treatment seeking behavior and attitudes. J Community Psychol (2010) 38(3):350–68. 10.1002/jcop.20368 21274407 PMC3026177

[27] Matthews AK Corrigan PW Smith BM Aranda F A qualitative exploration of African Americans’ attitudes toward mental illness and mental illness treatment seeking. Rehabil Educ (2006) 20(4):253–68. 10.1891/088970106805065331

[28] Hines-Martin V Malone M Kim S Brown-Piper A. Barriers to mental health care access in an African American population. Issues Ment Health Nurs (2003) 24:237–56. 10.1080/016128040390160775 12623684

[29] Jimenez DE Bartels SJ Cardenas V Alegría M Stigmatizing attitudes toward mental illness among racial/ethnic older adults in primary care. Int J Geriatr Psychiatry (2013) 28(10):1061–8. 10.1002/gps.3928 23361866 PMC3672370

[30] Givens JL Katz IR Bellamy S Holmes WC. Stigma and the acceptability of depression treatments among African Americans and Whites. J Gen Intern Med (2007) 22(9):1292–7. 10.1007/s11606-007-0276-3 17610120 PMC2219769

[31] Alvidrez J. Ethnic variations in mental health attitudes and service use among low-income African American, Latina, and European American young women. Community Ment Health J (1999) 35(6):515–30. 10.1023/A:1018759201290 10863988

[32] Nadeem E Lange JM Edge D Fongwa M Belin T Miranda J. Does stigma keep poor young immigrant and U.S.-born Black and Latina women from seeking mental health care? Psychiatr Serv (2007) 58(12):1547–54. 10.1176/appi.ps.58.12.1547 18048555

[33] Corrigan PW Edwards AB Green A Diwan SL Penn D. Prejudice, social distance, and familiarity with mental illness. Schizophr Bull (2001) 27:219–25. 10.1093/oxfordjournals.schbul.a006868 11354589

[34] Schnittker J Freese J Powell B Nature, nurture, neither, nor: black-white differences in beliefs about the causes and appropriate treatment of mental illness. Soc Forces (2000) 78(3):1101–32. 10.2307/3005943

[35] Feeg VD Prager LS Moylan LB Smith KM Cullinan M. Predictors of mental illness stigma and attitudes among college students: using vignettes from a campus common reading program. Issues Ment Health Nurs (2014) 35(9):694–703. 10.3109/01612840.2014.892551 25162192

[36] Wolff G Pathare S Craig T Leff J. Community knowledge of mental illness and reaction to mentally ill people. Br J Psychiatry (1996) 168(2):191–8. 10.1192/bjp.168.2.191 8837909

[37] Sánchez M Cardemil E Adams S Calista JL Connell J DePalo A Brave new world: mental health experiences of Puerto Ricans, immigrant Latinos, and Brazilians in Massachusetts. Cultur Divers Ethnic Minor Psychol (2014) 20(1):16–26. 10.1037/a0034093 24491125

[38] Plant EA Butz DA. The causes and consequences of an avoidance-focus for interracial interactions. Pers Soc Psychol Bull (2006) 32(6):833–46. 10.1177/0146167206287182 16648207

[39] Perry SP Dovidio JF Murphy MC van Ryn M. The joint effect of bias awareness and self-reported prejudice on intergroup anxiety and intentions for intergroup contact. Cultur Divers Ethnic Minor Psychol (2015) 21(1):89–96. 10.1037/a0037147 25111552 PMC4411950

[40] West K Hewstone M Lolliot S. Intergroup contact and prejudice against people with schizophrenia. J Soc Psychol (2014) 154(3):217–32. 10.1080/00224545.2014.888327 24873025

[41] Stathi S Tsantila K Crisp RJ. Imagining intergroup contact can combat mental health stigma by reducing anxiety, avoidance and negative stereotyping. J Soc Psychol (2012) 152(6):746–57. 10.1080/00224545.2012.697080 23057193

[42] Rao D Feinglass J Corrigan P. Racial and ethnic disparities in mental illness stigma. J Nerv Ment Dis (2007) 195:1020–3. 10.1097/NMD.0b013e31815c046e 18091196

[43] Interian A Martinez IE Guarnaccia PJ Vega WA Escobar JI. A qualitative analysis of the perception of stigma among Latinos receiving antidepressants. Psychiatr Serv (2007) 58(12):1591–4. 10.1176/appi.ps.58.12.1591 18048562 PMC2288553

[44] Dalky HF. Mental illness stigma reduction interventions: review of intervention trials. West J Nurs Res (2012) 34(4):520–47. 10.1177/0193945911400638 21389251

[45] Day EN Edgren K Eshleman A Measuring stigma toward mental illness: development and application of the mental illness stigma scale. J Appl Soc Psychol (2007) 37:2191–219. 10.1111/j.1559-1816.2007.00255.x

[46] Reynolds WM Development of reliable and valid short forms of the Marlowe–Crowne social desirability scale. J Clin Psychol (1982) 38(1):119–25. 10.1002/1097-4679(198201)38:1<119::AID-JCLP2270380118>3.0.CO;2-I

[47] Aiken LS West SG Multiple Regression: Testing and Interpreting Interactions. Newbury Park: SAGE Publications (1991).

[48] Broussard B Goulding SM Talley CL Compton MT Social distance and stigma toward individuals with schizophrenia: findings in an urban, African American community sample. J Nerv Mental Dis (2012) 200(11):935–40. 10.1097/NMD.0b013e3182718c1b 23124176

[49] Pedersen ER Paves AP. Comparing perceived public stigma and personal stigma of mental health treatment seeking in a young adult sample. Psychiatry Res (2014) 219(1):143–50. 10.1016/j.psychres.2014.05.017 24889842 PMC4086709

[50] Gary FA. Stigma: barrier to mental health care among ethnic minorities. Issues Ment Health Nurs (2005) 26:979–99. 10.1080/01612840500280638 16283995

[51] Interian A Lewis-Fernández R Dixon LB Improving treatment engagement of underserved U.S. racial-ethnic groups: a review of recent interventions. Psychiatr Serv (2013) 64(3):212–22. 10.1176/appi.ps.201100136 23203442

[52] Snowden LR African American service use for mental health problems. J Community Psychol (1999) 27(3):303–3019. 10.1002/(SICI)1520-6629(199905)27:3<303::AID-JCOP5>3.0.CO;2-9

[53] Sue S Zane N Young K Research on psychotherapy with culturally diverse populations. 4th ed In: Bergin AE Garfield SL, editors. Handbook of Psychotherapy and Behavior Change. New York: John Wiley & Sons (1995). p. 783–817.

[54] Corrigan PW Watson AC Warpinski AC Garcia G. Implications of educating the public on mental illness, violence and stigma. Psychiatr Serv (2004) 55:577–80. 10.1176/appi.ps.55.7.834-a 15128968

[55] Pettigrew TF Tropp LR. A meta-analytic test of intergroup contact theory. J Pers Soc Psychol (2006) 90(5):751–83. 10.1037/0022-3514.90.5.751 16737372

[56] Whaley AL Ethnic and racial differences in perceptions of dangerousness of person with mental illness. Psychol Serv (1997) 48(10):1328–1130. 10.1176/ps.48.10.1328 9323754

